# Gut Microbes Associated with Neurodegenerative Disorders: A Comprehensive Review of the Literature

**DOI:** 10.3390/microorganisms12081735

**Published:** 2024-08-22

**Authors:** Christos Koutsokostas, Ermis Merkouris, Apostolos Goulas, Konstantina Aidinopoulou, Niki Sini, Theofanis Dimaras, Dimitrios Tsiptsios, Christoph Mueller, Maria Nystazaki, Konstantinos Tsamakis

**Affiliations:** 1 Neurology Department, Democritus University of Thrace, 68100 Alexandroupoli, Greece; chriskoutsokostas2001@gmail.com (C.K.); ermmerk@gmail.com (E.M.); gapostolos2@gmail.com (A.G.); konstant2002aid@gmail.com (K.A.); nickysini@yahoo.com (N.S.); thiou295@gmail.com (T.D.); 2 3rd Neurology Department, Aristotle University, 54124 Thessaloniki, Greece; tsiptsios.dimitrios@yahoo.gr; 3 Institute of Psychiatry, Psychology and Neuroscience (IoPPN), King’s College London, London SE5 8AB, UK; christoph.mueller@kcl.ac.uk; 4 Biomedical Research Centre, South London and Maudsley NHS Foundation Trust, London SE5 8AF, UK; 5 2nd Department of Psychiatry, University General Hospital ‘Attikon’, 12462 Athens, Greece; nystazaki@yahoo.gr; 6 Institute of Medical and Biomedical Education, St George’s, University of London, London SW17 0RE, UK

**Keywords:** gut microbiome, dysbiosis, alterations, gut–brain axis, neurodegenerative, neuropsychiatric, Parkinson’s disease, Alzheimer’s disease

## Abstract

Evidence shows that neurodegenerative and neuropsychiatric disorders are influenced by alterations in the gut microbiome. Various diseases have been linked to microbiome dysbiosis, yet there are inconclusive data regarding which microorganisms are associated with each disorder. The aim of our study is to systematically review the recent literature of the past decade to clarify whether the gut microbiome contributes to the understanding of pathogenesis and progression of neurodegenerative disorders. Most included studies showed a strong correlation between the relative abundance of certain microorganisms, mainly species of the phyla *Firmicutes* and *Bacteroidetes*, and disorders such as Parkinson’s disease (PD) and Alzheimer’s disease (AD). It is speculated that the microorganisms and their byproducts have a significant role in brain protein accumulation, neuro-inflammation, and gut permeability. The estimation of microbial populations could potentially improve clinical outcomes and hinder the progression of the disease. However, further research is needed to include more diseases and larger patient samples and identify specific species and subspecies associated with these disorders.

## 1. Introduction

Neurodegenerative disorders comprise a large number of chronic illnesses, where neurons gradually lose their ability to function and eventually decay. The most common neurodegenerative disorders include Parkinson’s disease (PD), Alzheimer’s disease (AD), Huntington’s disease (HD) and multiple system atrophy (MSA). Given that the incidence of these conditions increases substantially with age, it is anticipated that the number of cases will continue to rise in the near future due to increased life expectancies in many countries. However, with few exceptions, the relative contributions of hereditary and environmental variables to the causation of these disorders are not fully understood [1]. At present, it is not feasible to cure or stop the progression of these disorders; however, on many occasions, their physical or mental symptoms can be alleviated by various treatments [2]. Neuropsychiatric disorders include a spectrum of neurobehavioral disorders and are a subset of mental disorders for which there is some understanding of the underlying brain pathology. These disorders range from serious mental illness such as schizophrenia to conditions such as HD and multiple sclerosis (MS) [3,4].

Due to similar signaling pathways, mechanisms, and, above all, therapeutic challenges, neurodegenerative and neuropsychiatric disorders are often regarded as “two sides of the same coin” [5,6]. Their various similarities provide confidence for current studies aimed at identifying medication that specifically target neurodegenerative and neuropsychiatric disorders [7]. Recent developments in translational research have focused on pharmaceutical treatments utilized in both of these types of disorders, which includes natural substances, multitarget drug ligands, non-coding RNAs, and micro-RNAs [8]. 

Alterations in the gut microbiome can influence cognitive and psychological functions through the microbiota–gut–brain (MGB) axis [9,10]. The term MGB refers to the complicated bidirectional interactions between the central nervous system (CNS) and the gastrointestinal (GI) tract; its main functions include monitoring gut activity as well as connecting the peripheral gut mechanisms, such as immune activation, gut permeability, and enteric reflex [11]. It is widely recognized that the intestinal microbiota is highly responsive to many environmental stimuli with variables such as age [12] and diet [13,14] playing a central role in determining the composition of the gut microbiome. Similarly, in neurodegenerative disorders such as PD, individuals may experience significant dietary changes and eating behavior problems [15] that can potentially lead to alterations in the composition of their gut microbiota [16]. Numerous studies have shown that degenerative diseases are functionally linked to dysbiosis of the human gut microbiota. Animal models have provided evidence for the potential importance of the gut microbiota in the pathogenesis of AD [17], and several recent studies have also confirmed this in humans [18,19]. The hypothesis that PD is associated with gut inflammatory reactions was first proposed by Braak et al. in 2003 [20]. Dysbiosis and gut inflammation in PD have also been demonstrated in studies on human [21] and animal subjects [22,23]. Remarkable changes in the composition of the gut microbiota have been observed in amyotrophic lateral sclerosis (ALS) mouse models [24,25] and in ALS patients compared to healthy controls [26,27]. In HD, there is limited evidence for the role of the gut microbiome; however, individuals with HD have been reported to have a lower abundance of *E. hallii*, correlated with more severe motor signs, and altered gut microbial composition compared to healthy controls [28].

Our review offers a comprehensive analysis of recent evidence linking gut microbiome dysbiosis to neurodegenerative and neuropsychiatric disorders, a rapidly evolving field with outstanding therapeutic potential. Contrary to the limited and often inconclusive research currently in existence, which often focus on specific diseases or present equivocal results, this study integrates data across multiple disorders reviewed in a comprehensive manner, emphasizing on similar microbial alterations and the potential impact on disease mechanisms. This integrative approach not only highlights the significance of the gut microbiome in the etiology of disorders like PD, AD, and MSA, but also emphasizes the distinct microbial signatures associated with each condition. Among several neurodegenerative and neuropsychiatric disorders, we chose to focus on these specific ones, because they not only affect millions of individuals but are also the most severe. Our findings lay the foundation for future research focused on developing individualized therapeutic strategies and microbiome-based diagnostics, which will ultimately lead to more efficient management and treatment of these debilitating diseases.

## 2. Materials and Methods

The Preferred Reporting Items for Systematic Reviews and Meta-Analyses (PRISMA) checklist was used to guide this study. Our study’s methods were a priori designed.

### 2.1. Search Strategy

Two databases (MEDLINE and Scopus) were selected for carrying out the present literature search, which was conducted by two investigators (A.G. and K.A.). To trace all relevant studies published between 1 January 2014 and 31 December 2023, the following keywords were used: “gut” AND “microbiome” OR “microbiota” AND “alterations” OR “changes” AND “neurodegenerative” OR “neuropsychiatric”. All the retrieved articles were also hand-searched for any further potential eligible articles. Any disagreement regarding the screening or selection process was solved by a third investigator (C.K.) until a consensus was reached.

### 2.2. Selection Criteria

Only full-text original articles published in the English language were included. Secondary analyses, reviews, guidelines, notes, errata, letters, meeting summaries, comments, unpublished abstracts, or studies conducted on animals were excluded. There was no restriction on study design or sample characteristics.

### 2.3. Data Extraction

Data extraction was carried out using a predefined data form created in Excel. We recorded the author, the year of publication, the type of study, the disease to which the article referred, the method of evaluation, the way the microbiome was identified, and finally the main findings of each study.

### 2.4. Data Analysis

No statistical analysis or meta-analysis was performed due to the high heterogeneity among the studies. Thus, the data were only descriptively analyzed.

## 3. Results

**Figure 1:**
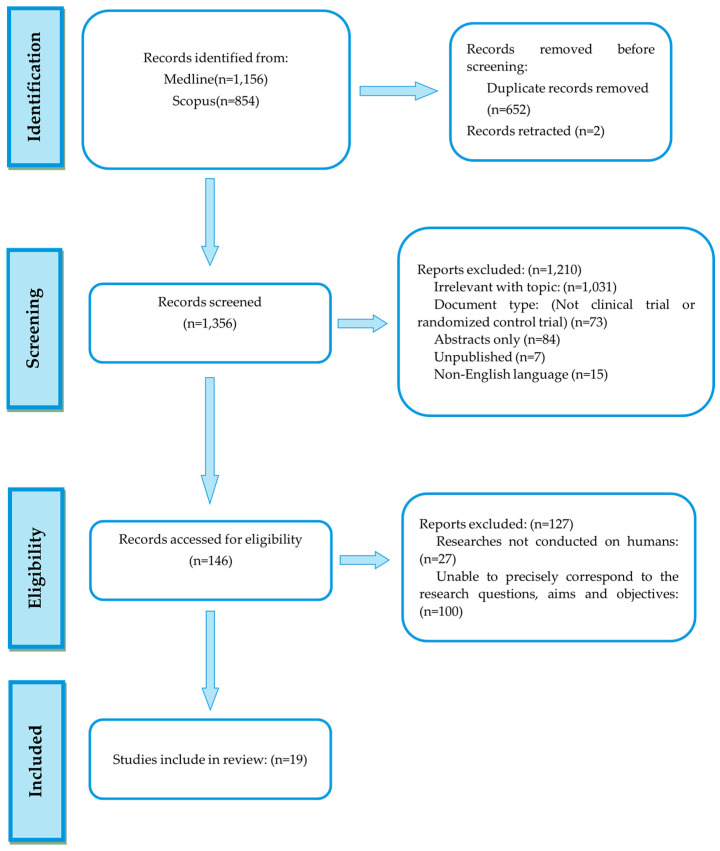
Flowchart of studies’ selection.

Overall, 2010 records were retrieved from the database search. Duplicates were removed; hence, a total of 1356 articles were selected. After dismissing irrelevant studies and screening the full texts of the articles, 19 studies were eligible for inclusion (Figure 1).

### 3.1. Type of Study

Out of 19 included studies, 13 were cohort studies, 4 were case–control studies, 1 was a randomized pilot clinical study, and 1 more was a two-sample bi-directional Mendelian randomized analysis (Table 1).

A summary of the types of studies included in our review can be seen in Table 1.

### 3.2. Related Disease

Nine studies focused on Parkinson’s disease, four reported on Alzheimer’s disease and three studies concerned Amyotrophic Lateral Sclerosis. There were also four more studies, each of which focused on one of the mentioned diseases: Creutzfeldt–Jakob disease, Huntington’s disease, multiple sclerosis, and multiple system atrophy.

The disorders reported in each study are summarized in Table 2.

### 3.3. Methods of Evaluation

Due to the multiple diseases studied in this paper, there were a variety of methods for assessing the disease and its severity. The most used ones were the Mini-Mental State Examination (MMSE)-seven studies (Parkinson’s disease, Alzheimer’s disease, Creutzfeldt–Jakob disease, and multiple system atrophy), Montreal Cognitive Assessment (MoCA)-five studies (Parkinson’s disease, Alzheimer’s disease, and Creutzfeldt–Jakob disease), Hoehn and Yahr Scale-four studies (Parkinson’s disease), Unified Parkinson’s Disease Rating Scale (UPDRS)-three studies (Parkinson’s disease), El Escorial Criteria-three studies (Amyotrophic Lateral Sclerosis), Hamilton Depression Rating Scale (HAMD)-two studies (Parkinson’s disease), Hamilton Anxiety Rating Scale (HAMA)-two studies (Parkinson’s disease). In addition, other twenty-four scales were used in one study to evaluate the discussed disease; some examples are the 6-Minute Walk Test (6MWT) and Modified Fatigue Impact Scale (MFIS-5) for multiple sclerosis, Unified Huntington’s Disease Rating Scale (UHDRS) for Huntington’s disease, and Unified Multiple System Atrophy Rating Scale (UMSARS) for multiple system atrophy. Paraclinical and laboratory tests were also used to assess the evaluation in some studies. Table 3 summarizes all scales and tests utilized in the included studies.

### 3.4. Estimation of Microbiome

For the estimation of microbiome, all studies included fecal sample collection, stool analysis, and DNA extraction. Furthermore, most of them used metagenomic sequencing libraries for different regions of the bacterial 16S rRNA gene, while only one of them was used for different regions of eukaryotic 18S rRNA genes. Finally, all of them included PCR amplification.

### 3.5. Main Findings

The main findings of the included studies are summarized in Table 4.

The demographic and clinical parameters of the participants in the included studies are summarized in Supplementary Table S1.

#### 3.5.1. Parkinson’s Disease

The intestinal microbiota of patients with PD proved to be more complex and diverse as evidenced by the alpha diversity indices of the fecal microbiota which were significantly greater in these patients than in healthy controls [29]. Additionally, the beta diversity index, an indicator of similarity/dissimilarity between microbiome pairs [44], (e.g., how different the diversity of bacteria between healthy controls is compared to diseased individuals [9]) showed a substantial qualitative difference between PD patients and controls [29]. Similar results were observed by Li et al. [30]. In addition, the percentages of genera *Holdemania*, *Anaerotruncus*, *Clostridium IV*, *Clostridium XVIII*, *Sphingomonas*, *Aquabacterium*, and *Butyricicoccus* differed significantly between the patient and healthy groups, indicating a possible connection between these genera and PD [22]. A correlation between some species and clinical features of PD was also found. For instance, there was a negative correlation between the genus *Bifidobacterium* and HAMD scores, indicating that decreased *Bifidobacterium* levels were linked to PD’s depression symptoms [30]. Moreover, the genera *Dorea* and *Phascolarctobacterium* showed a negative correlation with levodopa (L-dopa)-equivalent doses, suggesting that the microbiota may influence drug metabolism and vice versa [29]. Nevertheless, Weis et al. found that among PD patients receiving L-dopa treatment, there was a relative increase in the *Clostridium* cluster XI and its related members *Peptoniphilus* and *Finegoldia* [34]. Since both genera can ferment peptones and amino acids, it is possible that they contribute to the degradation of L-dopa, thus diminishing its effectiveness [34].

Hegelmaier et al., among others, observed the significance of short-chain fatty acids (SCFAs) as a potential biomarker in neurodegenerative diseases [37]. SCFAs are a metabolic product of some intestinal bacteria and are mainly produced through fermentation by the families *Lachnospiraceae* and *Prevotellaceae* as well as the genera *Blautia*, *Akkermansia*, *Faecalibacterium*, and *Roseburia* in a high-fiber diet [37]. With the exception of the genus *Akkermansia*, the abundance of all SCFA producers decreased in PD. SCFAs including acetate, butyrate, and propionate are vital for the body’s immunological functions, the intestinal barrier integrity, as well as the regulation of the enteric nervous system (ENS) [37]. SCFAs’ effects on inflammatory processes in the intestine may indirectly affect permeability and possibly result in the secretion of alpha synuclein (α-Syn) in the intestinal wall. The decreased abundance of SCFA producers, such as the families and the genera mentioned above, could therefore be related to reduced SCFA levels in PD [37].

Vascellari et al. noted that the relative abundance of *Firmicutes* taxa decreased significantly in PD patients, especially the *Lachnospiraceae* family and other important genera like *Blautia*, *Coprococcus*, and *Butyrivibrio* [35]. A number of *Lachnospiraceae* family members are gaining increasing interest due to their ability to produce SCFAs, which may play a role in the development of GI motility disorders [35]. Similarly, a decrease in *Fusicatenibacter*, *Faecalibacterium*, and *Prevotella* was observed, among other bacterial taxa, which are thought to have neuroprotective, health-promoting, and anti-inflammatory properties or exerting additional beneficial effects on the epithelial barrier [34,37]. The reduction in butyrate-producing genera was observed in PD patients who had higher levels of calprotectin [37]. Calprotectin is an inflammatory indicator of alterations in the gut microbiota linked to low-grade, asymptomatic inflammation [37].

Inspired by an animal study conducted by Sampson et al. [45] on the involvement of *Akkermansia muciniphila* and *Bilophila wadsworthia* in microbial populations that contribute to the deterioration of motor symptoms in genetically predisposed mice, Hertel et al. confirmed that both of these species are more abundant in PD microbiomes [36], thus supporting the connection to the intensity of clinical symptoms in PD patients. Specifically, *Bilophila wadsworthia* has been linked to intestinal inflammation and is an essential species for sulfite synthesis in the gut microbiome [36]. Sulfite is a documented neurotoxin that lowers glutathione levels in neurons, hence impacting brain mitochondrial energy homeostasis in PD patients [36]. On the other hand, *Akkermansia muciniphila* produces hydrogen sulfide (H2S), which also contributes to the pathophysiology of PD. H2S is a highly reactive signaling molecule with multiple functions throughout the body and is a pro-inflammatory agent that threatens the structural integrity of the intestinal mucus layer [36]. For this reason, a greater abundance of this species increases intestinal H2S levels and may be responsible for not only PD-related GI symptoms, such as constipation, but increased absorption of bacterial toxins through a weakened intestinal barrier [36].

Based on research by Murros et al. [33], certain species of *Desulfovibrio* bacteria (DSV) were detected in the majority of PD patients’ stool samples, while the percentage of DSV correlated with the severity of the disease [33]. DSV permanently colonizes the GI tract, multiplies, and generates elevated levels of H2S that surpass the ability of the intestinal mucosa to detoxify high levels of hydrogen sulfide [33]. Besides that, they synthesize lipopolysaccharides (LPSs) and stimulate α-Syn oligomerization and its accumulation in the intestinal enteroendocrine cells. Toxic α-Syn oligomers propagate in a prion-like way, primarily via the vagus nerve, but also via the bloodstream, from the enteroendocrine cells to the brain, where they ultimately damage the brain’s dopaminergic system [33]. It was found that the magnetite nanoparticles synthesized by DSV may enter the circulation from the GI tract, transcend the blood–brain barrier, and hasten the accumulation of α-Syn in the brain [33]. The above reasons suggest that DSV may play a major role in the pathophysiology of PD, while also hinting potential therapeutic targets, i.e., products of PD-associated DSV [33].

Among the several microbiome alterations reported, the observation of a decrease in the relative abundance of *Dolichospermum*, a member of the phylum *Cyanobacteria*, in PD patients is particularly noteworthy [35]. Interestingly, a number of neurotoxins produced by *Cyanobacteria* are thought to be responsible for the protein misfolding and aggregation phenomena observed in PD and other neurodegenerative diseases [35]. Therefore, this finding is rather peculiar given the unclear role of *Dolichospermum*; hence, the decrease in the abundance of *Dolichospermum* in PD patients deserves further investigation in regard to its potential role in the pathophysiology of PD [35].

Out of seven genus-equivalent fungal taxa, only *Geotrichum* had a noticeably greater relative abundance [31]. One may hypothesize that rather than real depletion, the apparent decrease in the other less-abundant taxa is merely the result of *Geotrichum* overgrowth [31]. The authors speculate that in addition to *Geotrichum*, there may be microbiome differences in other eukaryotic taxa between PD patients and controls. Despite not being considered typical members of the human gut microbiota, bacterivorous gliding Zooflagellates including *Heteromita*, *Cercomonas*, and *Poterioochromonas* may influence the pathogenicity, metabolism, and morphology of intestinal bacteria by selective protistan grazing [31]. It is widely recognized that these fungi produce a variety of toxins and affect the mucosal cytokine response, which alters the GI homeostasis and the composition of the bacterial gut microbiota [31].

*Ruminococcaceae* abundance was observed by Hegelmaier et al. [37] to positively correlate with the Unified Parkinson’s Disease Rating Scale III (UPDRS III) [37]. On top of that, the proportion of *Ruminococcaceae* increases with the duration of the disorder [46]. The fact that the relative abundance of UPDRS III increases as the disorder advances offers various, potential possibilities for future therapeutic options [46].

**Figure 2:**
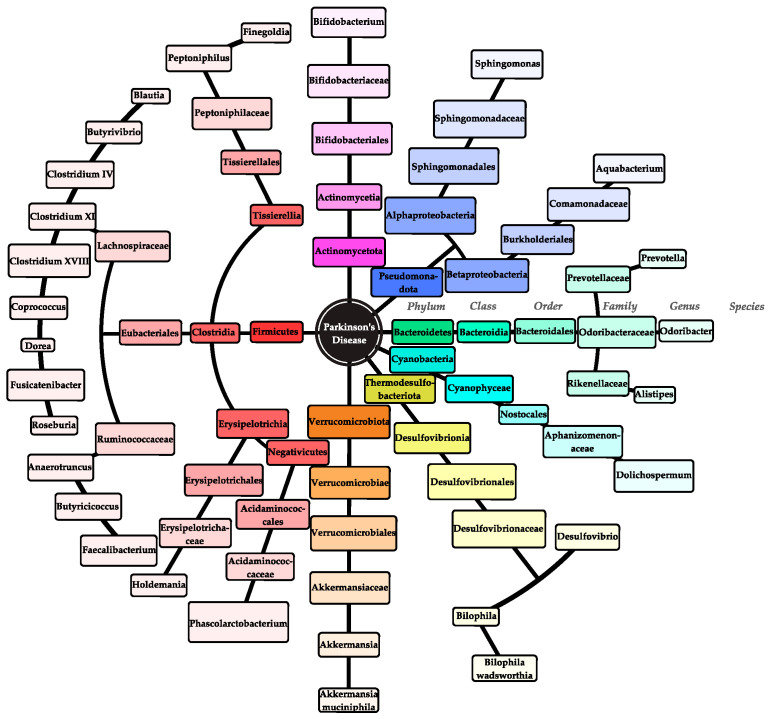
All bacterial microorganisms (from entry to periphery: phylum–class–order–family–genus–species) related to PD.

Taxonomy network diagrams from a study by Li et al. [30] revealed that while microorganisms in different samples exhibited different levels of adaptability, their interactions within a given sample were quite similar [30]. It is noteworthy that certain phyla or genera that differed significantly between the control and patient groups formed subnetworks [30]. For instance, a subnetwork of unidentified *Alistipes*, *Odoribacter*, and *Ruminococcaceae* of the phyla *Bacteroidetes* and *Firmicutes* emerged, whose abundance was proven to be altered in PD patients and has been previously correlated with mild cognitive impairment [30]. The synergistic behavior of these bacteria could play an essential role in the progression of PD and offer an alternative perspective for studying the composition and function of complex microbial ecosystems [30]. The microbes involved in PD are illustrated in Figure 2 and Supplementary Figure S1.

#### 3.5.2. Alzheimer’s Disease

AD patients with dementia and amnestic mild cognitive impairment (aMCI) had a microbiome composition that differed significantly from the controls [19]. By identifying the predominant microbiota, it is possible to differentiate aMCI and AD from the controls, as well as AD from aMCI [19]. Specifically, patients with aMCI had a significantly greater relative abundance of *Bacteroidetes* compared to patients with AD and healthy individuals [19]. As a result, a noteworthy association was found between *Bacteroidetes* and MMSE cognitive impairment scores, suggesting a possible involvement of *Bacteroidetes* in the predementia phase of AD [19]. Additionally, proportions of pro-inflammatory *Gammaproteobacteria*, *Enterobacteriales*, and *Enterobacteriaceae* of the phylum *Proteobacteria* gradually increased from controls to prodromal aMCI and AD stages. These particular alterations showed a strong correlation with the clinical severity of AD [19]. More importantly, the *Enterobacteriaceae*-based models were able to accurately discriminate AD from both aMCI and the controls [19]. Members of the *Enterobacteriaceae* family, particularly *Escherichia coli* (*E. coli*), are thought to be pro-inflammatory bacteria [19], with their primary endotoxin, LPS, having been detected in large quantities in the perinuclear area as well as in the neocortex and hippocampal regions of postmortem AD patients’ brains. In a similar manner, Raghavan et al. noticed that *Enterobacteriaceae* and *E. coli* express a protein called amyloid curli, which shares properties with immunomodulatory and pathological human amyloids including α-Syn and serum amyloid A linked to neuro-inflammation [32]. Curli stimulates the build-up of insoluble amyloid clusters of α-Syn protein, leading to inflammation and neuronal degradation typical of Lewy body-associated synucleinopathies such as AD and PD [32].

Liu et al. established that the abundance of SCFA producers, such as *Ruminococcus*, *Lachnospiraceae*, and *Clostridiaceae* from the *Firmicutes* phylum, was considerably lower in AD patients and was favorably correlated with indices of the disease’s cognitive status, such as MMSE and MoCA scores [19]. *Ruminococcus* reduction may result in diminished intestinal permeability, allowing gut amyloid to more easily enter the bloodstream and subsequently build up in the brain. Similarly, Zhou et al. noted a decrease in the proportions of *Eubacterium*, *Odoribacter*, *Papillibacter*, and *Anaerobacterium* [39]. Consequently, there were more SCFA-producing bacteria in the gut microbiota of healthy individuals, which may potentially play a role in preventing the development of AD [39].

Finally, elevated levels of the gut microbial neurotransmitter GABA, which is a downstream product of *Blautia*-dependent arginine metabolism, were possibly linked to decreased risk of AD, as demonstrated by Zhuang et al. [38]. It has been proven that GABA, a key inhibitory neurotransmitter in the human CNS, influences neurological processes and cognition, while also being crucial for microbiota-host communication in behavior and brain function [38]. GABAergic activities may be a crucial element in the overall process of AD pathogenesis, which appeared to be more resilient to neurodegenerative alterations in the aged brain [38]. The microbes involved in AD are illustrated in Supplementary Figure S2.

#### 3.5.3. Amyotrophic Lateral Sclerosis

Parallel to other neurodegenerative disorders such as AD and PD, dysbiosis in the gut microbiome of ALS patients was detected by Zeng et al. [26]. Specifically, they indicated that the relative abundance of *Bacteroidetes* at the phylum level was greater in patients than in healthy individuals, while at the same time showing a reduced ratio of *Firmicutes* at the phylum level and *Megamonas* at the genus level [26]. It is possible that the reduced abundance of *Firmicutes* and increased richness of *Bacteroidetes*, especially the decreased *Firmicutes/Bacteroidetes* ratio in the ALS group, reflects the deteriorated condition of ALS patients and may serve as a dysbiosis biomarker for the disorder [26]. Yet again, the underlying reason is that the production of SCFAs assists in the reduction in the excessive accumulation of mutated proteins in human intestinal epithelial cells [26]. In a similar way, Nicholson et al. [27] reported a decreased relative abundance of *Eubacterium rectale* and *Roseburia intestinalis*, two major species that produce butyrate, in ALS patients. Given that butyrate and other SCFAs can inhibit NF-jB activation and encourage the differentiation of regulatory T-cells, they may have an impact on systemic inflammation [27]. It has been established that abnormalities in these two inflammatory pathways have a role in ALS pathogenesis [27].

Conversely, Brenner et al. found no evidence linking ALS to a significantly altered composition of the gut microbiota [40]. Notably, the authors found no significant differences in the diversity, quantity, and relative abundance of the fecal microbiota as well as in the anticipated metagenomes between ALS patients and control subjects, with the exception of varying proportions of uncultured *Ruminococcaceae* at the genus level, which account for 1 of the 336 microbial species examined [40]. Bacteria involved in ALS are illustrated in Supplementary Figure S3.

#### 3.5.4. Multiple System Atrophy

Wan et al.’s study revealed that MSA patients presented a microbiota composition identical to that in PD, but also somewhat unique, consisting of a high abundance of the genus *Akkermansia* and the species *Roseburia hominis*, *Akkermansia muciniphila*, and *Staphylococcus xylosus* yet a low abundance of genera *Blautia*, *Bifidobacterium*, *Aggregatibacter and Megamonas* as well as the species *Clostridium nexile*, *Phocaeicola coprocola*, *Megamonas funiformis and Phocaeicola plebeius* [41]. Remarkably, *Akkermansia* exhibits pro-inflammatory qualities, enhancing genes associated with the antigen presentation process, B and T cell receptor signaling, and IL-4 activation. This may be related to its ability to disrupt host mucus homeostasis, resulting in the collapse of the intestinal barrier [41]. Regarding the genera *Blautia* and *Bifidobacterium*, both are SCFA producers and have anti-inflammatory properties [41]. Inflammation plays a vital role in the development of MSA, and intestinal inflammation was proven to increase the risk of this disease [47,48]. Bacteria involved in MSA are illustrated in Supplementary Figure S4.

#### 3.5.5. Creutzfeldt–Jakob Disease

In CJD patients, two crucial microbial phyla, *Actinomycetota* and *Fusobacteriota*, were significantly elevated compared to the control group [43]. The proportion of the phylum *Actinomycetota* and its class *Actinomycetia*, along with its family *Bifidobacteriaceae*, increased, while in the phylum *Fusobacteriota*, the increase was noticed in the relative abundance of its class *Fusobacteriia*, the family *Fusobacteriaceae*, and the genus *Fusobacterium* [43]. The high relative abundance of *Actinomycetia* has also been found in AD and has been demonstrated to synthesize SCFA [43]. As mentioned above, SCFA in reasonable quantities can have certain benefits for the intestinal wall; however, a large excess of it may have detrimental effects [43]; for instance, a very low concentration of butyrate decreases the gut permeability, whereas a high concentration of butyrate causes gut epithelial apoptosis [43]. Bacteria involved in CJD are illustrated in Supplementary Figure S5.

#### 3.5.6. Huntington’s Disease

A number of physical manifestations or neurological changes observed in HD may be related to gut-driven regulation of brain inflammatory pathways, involving communication across the gut, endocrine, immunological, and neuronal pathways [49,50,51]. The richness and structure of the microbiome of HD patients were similar to other neurodegenerative diseases, including PD, AD, and ALS [50]. However, a study by Wasser et al. did not observe the previously reported increase in the proportion of *Bacteroidetes* and the decrease in *Firmicutes* [28]. Their finding that the abundance of *Firmicutes*, *Lachnospiraceae*, and *Akkermansiaceae* is significantly reduced in HD patients is particularly interesting, since it is affiliated with inflammatory processes [51]. This could be due to *Akkermansiaceae* maintaining the intestinal barrier, as well as *Lachnospiraceae* and *Firmicutes* producing butyric acid, which reduces inflammation [51]. Bacteria involved in CJD are illustrated in Supplementary Figure S6.

#### 3.5.7. Multiple Sclerosis

Barone et al. [42] found that *Collinsella* and *Prevotella*, two bacteria associated with autoimmune diseases and elevated levels of the pro-inflammatory cytokine IL-17A, were more abundant in the microbial ecosystem of MS patients [42]. The overabundance of *Collinsella* has been shown to intensify the symptoms in MS by triggering pro-inflammatory responses and compromising the barrier stability, which in turn aggravates a chronic inflammatory response [42]. *Prevotella* has also been linked to inflammatory conditions, including rheumatoid arthritis, where it maintains an inflammatory state by stimulating pro-inflammatory cytokines (TNF-α and IFN-γ) produced by CD8+ T cells and TH17 [42]. Aside from that, the authors discovered a decreasing pattern for the bacterial genus *Eggerthella*, which is considered a possible biomarker for patients with autoimmune diseases such as MS and rheumatoid arthritis [42]. Bacteria involved in CJD are illustrated in Supplementary Figure S7.

Supplementary Table S2 summarizes all gut microbes associated with each neurodegenerative disorder.

## 4. Discussion

The majority of the studies included in our review focused on PD and on how the gut microbiome contributes to the development and progression of the disease. We observed many similarities in the mechanisms between PD and other synucleinopathies such as AD and MSA, as well as other neurodegenerative disorders such as ALS, HD, CJD, and MS.

Our findings indicate that fluctuations (increase or decrease in relative abundance) of certain microbial populations could be associated with some neurogenerative disorders. By examining a plethora of microorganisms, we concluded that the most notable change between patients and healthy controls involved primarily the phyla *Firmicutes* and *Bacteroidetes*, as well as the phyla *Pseudomonadota*, *Actinomycota*, and *Verrucomicrobiota*. More specifically, alterations in members of the *Lachnospiraceae*, *Ruminococcaceae*, and *Enterobacteriaceae* families were associated with the course, severity, and prognosis of these disorders. On another note, we found that specific microbial genera could be associated with certain symptoms, as indicated by the combination of relative abundance of microorganisms with clinical scores and scales (UPDRS III, HAMD, MMSE, etc.) [37]; however, these findings need to be supported by further wide and comprehensive studies. Nevertheless, these findings could be potentially useful in future clinical practice, in assessing the stage of a particular disease as well as assisting in the differential diagnosis between related conditions [19].

Among various different mechanisms, we concluded that SCFAs play a major role in the pathogenesis of neurodegenerative disorders. As a metabolic product of many microbes found in the intestines, they are vital for the body’s immunological functions, the intestinal barrier integrity as well as the regulation of the ENS [19,39,43]. In these disorders, the relative abundance of SCFA producers such as *Blautia*, *Ruminococcus*, *Roseburia*, and *Fusicatenibacter* of the phylum *Firmicutes* and *Prevotella* and *Odoribacter* of the phylum *Bacteroidetes* decreased, indicating a reduction in SCFAs and a lack of their beneficial properties [26,37]. In PD and AD, depletion of SCFAs promotes intestinal inflammation caused by the production of pro-inflammatory cytokines that diminishes intestinal permeability, thus leading to α-Syn secretion, its traversal, and its accumulation in the brain [27,34,35]. This is in accordance with a review about AD by Jiang et al., which states that bacteria populating the intestines can secrete large amounts of amyloids and LPSs, contributing to the modulation of signaling pathways in the brain [52]. Although *Akkermansia* is a SCFA producer, the increased relative abundance of *Akkermansia* has been found to harm the intestinal barrier and increase the absorption of bacterial toxins [28,37]. This is due to H2S production and the promotion of inflammation through cytokine signaling [36,41]; its harmful effects outweigh the benefits of SCFAs, as confirmed by Cani et al.’s review [53]. In addition, we highlighted the pro-inflammatory effect of *Enterobacteriaceae*, with emphasis on *E. coli*, especially through the presence of LPS, as well as the expression of amyloid curli, which is associated with neuro-inflammation in synucleinopathies [19,32]. Specifically in MS, the species *Collinsella*, *Prevotella*, and *Eggerthella* may serve as a potential biomarker for autoimmune disorders, since they are associated with IL-17A production and chronic inflammation [42,54]. The concept of microbiota-induced neuro-inflammation is strongly supported by Quigley et al. [55] and Chen et al. [56], stating that cytokines in the bloodstream and bacteria-derived molecules impair blood–brain barrier function and trigger an inflammatory response.

Our results indicate that microorganisms play a significant role in the development and pathophysiology of neurodegenerative diseases. Furthermore, gut microbiome alterations pave the way for future prognostic markers and new therapeutic targets [42]. In particular, we found that the ratio between the relative abundance of two phyla can act as a potential dysbiosis biomarker, which may prove to be a valuable tool for clinical practice [26,37]. Understanding the newly discovered pathophysiology of these disorders opens up new therapeutic possibilities, as it enables the creation of individualized treatment options that focus on specific parts of the pathway [33]. For instance, by increasing the relative abundance of a species with protective properties, such as SCFA producers, the clinical symptoms and the progression of the disease may improve [37,46].

## 5. Limitations and Future Directions

Our study has several limitations. The majority of studies were conducted in a single center, and sometimes, the results presented were based on a small sample size. Therefore, future research should include larger populations of patients suffering from a specific neurodegenerative disorder. Also, the population under investigation exhibited significant heterogeneity, with several studies offering inadequate or no information on patient treatment and relevant confounders including the duration of the patient’s condition. Additionally, some findings might also be caused by different food preferences of the investigated patients, although all of them reported an omnivorous European diet.

Looking at the future, further analyses are needed to verify whether the observed differences in community composition might be of biological/medical relevance especially in the case of PD. To do so, more complex analyses using multiple variable regions of the 18S rRNA gene sequence and ITS region sequences or even a metagenomics approach will be needed. Since only 0.01% of metagenomic sequences generated from human gut samples can be aligned to fungal genomes, such metagenomics approaches require sufficient sequencing depth. However, the taxonomic resolution of this approach may be insufficient for determining the relative abundance of the butyrate-producing species that we found to be depleted in individuals with ALS, as demonstrated by the fact that, using data from 16S sequencing alone, we could not demonstrate significant differences between ALS cases and controls in our own study. Altered diversity of the gut microbiota across these diseases indicates that gut dysbiosis appears to be a common feature of at least some neurodegenerative diseases. “Microbiome intervention, aiming at regulating the mediators of microbiota–gut–brain communication affected by microbial metabolism such as SCFAs and serotonin, might provide a new therapeutic option for AD” [19]. These changes in the gut bacteria could be an important part of ALS pathophysiology, might have a role as a disease biomarker, and could even act as a therapeutic target. However, further research is required, in order to include additional disorders, larger patient samples. and more taxonomically specific species.

## 6. Conclusions

In conclusion, gut microbiome alterations play a pivotal role in the presentation and development of neurodegenerative disorders. Fluctuations in microbial populations, particularly within certain families and phyla, have been reported to correlate with the course of the disease. Despite the limited data in this field, the results suggest promising avenues for future research as well as individualized treatment strategies. The latter could modify microbial populations and influence their metabolites in order to improve clinical outcomes and impede disease progression.

## Figures and Tables

**Table 1:** Types of included studies.

Type of Study	*n*
Cohort study	13
Case–control study	4
Randomized pilot clinical study	1
Two-sample bi-directional Mendelian randomization analysis	1

**Table 2:** Related disorders and authors on included studies.

Disease	n	Authors
Parkinson’s disease	9	Qian et al. (2018) [29], Li et al. (2022) [30], Weis et al. (2021) [31], Raghavan et al. (2023) * [32], Murros et al. (2021) [33], Weis et al. (2019) [34], Vascellari et al. (2020) [35], Hertel et al. (2019) [36], Hegelmaier et al. (2020) [37]
Alzheimer’s disease *	4	Liu et al. (2019) [19], Zhuang et al. (2020) ** [38], Raghavan et al. (2023) * [32], Zhou et al. (2021) [39]
Amyotrophic lateral sclerosis	3	Zeng et al. (2020) [26], Brenner et al. (2017) [40], Nicholson et al. (2020) [27]
Multiple system atrophy	1	Wan et al. (2019) [41]
Multiple sclerosis	1	Barone et al. (2021) [42]
Huntington’s disease	1	Wasser et al. (2020) [28]
Creutzfeldt–Jakob disease	1	Guo et al. (2022) [43]

* Raghavan et al.’s study included both Parkinson’s disease and Alzheimer’s disease. ** In one of them, schizophrenia was studied together with Alzheimer’s disease.

**Table 3:** Scales and tests utilized in the included studies.

Evaluation	n	Disease	Evaluation	n	Disease
MMSE	7	PD, AD, CJD, MSA	Laboratory tests	1	AD
MoCA	5	PD, AD, CJD	Hopkins Verbal Learning Test-Revised	1	HD
Hoehn and Yahr Scale	4	PD	HD-CAB	1	HD
UPDRS	3	PD	CAP	1	HD
El Escorial Criteria	3	ALS	Mass spectrometry	1	PD
NMS-Quest	2	PD	MDS-UPDRS	1	PD
HAMD	2	PD	MFIS-5	1	MS
HAMA	2	PD	6MWT	1	MS
UHDRS	1	HD	FFQ	1	MS
UMSARS	1	MSA	GC-MS analysis	1	PD
Trail Making Test	1	HD	Emotion Recognition Task	1	HD
Symbol Digit Modalities Test	1	HD	MRI	1	AD
Paraclinical tests	1	MS	CSF evaluation	1	CJD
Paced tapping	1	HD	CDR-SB	1	CJD
OTS of Cambridge	1	HD	CDR	1	AD
NPI	1	AD			

**Table 4:** Summary of included studies and main findings.

a/a	Articles/Authors	Type of Study	Related Disease	Method of Evaluation	Estimation of Microbiome-Sequencing Approach	Main Findings
1	Qian et al. (2018) [29]	Case–control study	Parkinson’s disease	Hoehn and Yahr Scale, UPDRS total, Part III scores, NMS-Quest, HAMA, HAMD, MMSE, MoCA	Amplification of the V3-V4 region of the bacterial 16S rRNA gene	The relative abundance of the genera *Clostridium IV*, *Aquabacterium*, *Holdemania*, *Sphingomonas*, *Clostridium XVIII*, *Butyricicoccus*, and *Anaerotruncus* increased in the feces of PD patients. The genera *Escherichia/Shigella* were negatively associated with disease duration. *Aquabacterium*, *Peptococcus*, and *Sphingomonas* in feces were associated with motor complications. The genera *Butyricicoccus* and *Clostridium XlVb* were associated with cognitive impairment.
2	Wan et al. (2019) [41]	Cohort study	Multiple system atrophy	UMSARS, MMSE	Metagenomic sequencing libraries	The gut microbiota of MSA patients were characterized by increased proportions of the genus *Akkermansia* and the species *Roseburia hominis*, *Akkermansia muciniphila*, *Alistipes onderdonkii*, *Streptococcus parasanguinis*, and *Staphylococcus xylosus*, and decreased proportions of the genera *Megamonas*, *Bifidobacterium*, *Blautia*, and *Aggregatibacter* and the species *Bacteroides coprocola*, *Megamonas funiformis*, *Bifidobacterium pseudocatenulatum*, *Clostridium nexile*, *Bacteroides plebeius*, and *Granulicatella adiacens*.
3	Liu et al. (2019) [19]	Cohort study	Alzheimer’s disease	MMSE, MoCA, MRI, laboratory tests	Amplification of the V3-V4 region of the bacterial 16S rRNA gene	The proportion of phylum *Firmicutes* significantly reduced (decreased abundance of the families *Clostridiaceae*, *Lachnospiraceae*, and *Ruminococcaceae* and the genera *Blautia* and *Ryminococcus*), whereas *Proteobacteria* was highly enriched in AD patients. *Gammaproteobacteria*, *Enterobacteriales*, and *Enterobacteriaceae* showed a progressive enriched prevalence from healthy controls to AD patients. The family *Enterobacteriaceae* was positively associated with the severity of AD. The relative abundance of *Bacteroidetes* was significantly enriched in the pre-onset stage of AD and unexpectedly decreased in the AD group to the control group.
4	Li et al. (2022) [30]	Case–control study	Parkinson’s disease	Hoehn and Yahr Scale, NMS, HAMD, HAMA, MoCA, MMSE, MDS-UPDRS	PCR amplification of the V5-V6 regions	Significant differences were found in microbiota composition of the gut between PD patients and healthy controls after adjusting for age, gender, and body mass index (BMI). The taxa class *Clostridia*, order *Clostridiales*, and family *Ruminococcaceae* in the gut microbiota were associated with weight and MMSE score.
5	Weis et al. (2021) [31]	Cohort study	Parkinson’s disease	N/A	Sequencing library for the V6-V7 regions of eukariotic 18S rRNA genes, PCR amplification	The proportions of the genera *Aspergillus*, *Cercomonas*, and *Heteromita*, as well as three unknown genus equivalent features of the phylum division *Charophyta*, the order *Chromulinales*, and the clade *Opisthokonta*, significantly decreased in PD patients. The relative abundance of genus *Geotrichum* significantly increased in PD patients.
6	Zhuang et al. (2020) [38]	Two-sample bi-directional Mendelian randomization analysis	Alzheimer’s disease, schizophrenia	N/A	Bacterial 16S rRNA gene sequencing	There was an increase in the relative abundance of *Blautia* associated with risk of AD. Elevated levels of the gut metabolite GABA were associated with a lower risk of AD. The increased proportion of the *Enterobacteriaceae* family and *Enterobacteriales* order were potentially related to a higher risk of schizophrenia (SCZ), while the *Gammaproteobacteria* class was associated with a lower risk of SCZ. Gut production of serotonin was potentially associated with a higher risk of SCZ. The increased relative abundance of the *Bacilli* class was associated with a higher risk of MDD. There was a lower relative abundance of the *Erysipelotrichaceae* family, the *Erysipelotrichales* order, and the *Erysipelotrichia* class and a higher relative abundance of unclassified *Porphyromonadaceae* in AD patients. MDD was related to increased proportion of unclassified *Clostridiales*, *OTU16802 Bacteroides*, and unclassified *Prevotellaceae*. SCZ was associated with an increased percentage of *OTU10589* unclassified *Enterobacteriaceae* and decreased proportion of unclassified *Erysipelotrichaceae*.
7	Raghavan et al. (2023) [32]	Randomized pilot clinical study	Alzheimer’s disease, Parkinson’s disease	N/A	Metagenomic sequencing libraries, Polymerase Chain Reaction (PCR)	The phylum *Firmicutes* was the most abundant followed by *Bacteroidetes*. The abundance of *Enterobacter* decreased to almost zero, while the abundance of *Prevotella* increased in ASD patients. The abundance of *Lactobacillus* and *Escherichia coli* decreased in ASD patients. The proportions of *Blautia* spp., *Coprobacillus* sp. and several *Clostridium* spp. decreased.
8	Guo et al. (2022) [43]	Cohort study	Creutzfeldt–Jakob disease	CSF evaluation, MMSE, MoCA, CDR-SB	PCR amplification of the V3-V4 regions of bacterial 16S rRNA genes	It was found that at the phyla level, the relative abundance of *Actinobacteria* and *Fusobacteria* significantly increased in the CJD group. At the class level, there was a significant enrichment of *Fusobacteriia*, *Actinobacteria*, and *Alphaproteobacteria* in the CJD group. However, it was found that the abundance of *Negativicutes* decreased in the CJD group compared to healthy controls. At the family level, significant increases were found in the proportions of *Fusobacteriaceae*, *Bifidobacterium*, *Succinivibrionaceae*, and *Enterococcaceae* within the CJD group. At the genus level, *Fusobacterium*, *Succinivibrio*, *Enterococcus*, and *Ruminococcus gnavus* groups and *Tyzzerella 4* were present at significantly higher levels in the CJD group, while the abundance of *Coprococcus 1*, *Lachnospiraceae*_ND3007, *Pseudobutyrivibrio*, *Roseburia*, and *Holdemanella* decreased in the CJD group.
9	Murros et al. (2021) [33]	Cohort study	Parkinson’s disease	Hoehn and Yahr Scale, MMSE	Polymerase Chain Reaction (PCR), bacterial 16S rRNA gene sequencing, HydA gene sequencing	It was found that all PD patients harbored *Desulfovibrio* bacteria in their gut microbiota, and the abundance of these bacteria increased in PD patients compared to control/healthy cohort. The concentration of *Desulfovibrio* species was correlated with the severity of PD.
10	Weis et al. (2019) [34]	Cohort study	Parkinson’s disease	Hoehn and Yahr Scale	Sequencing of the bacterial 16S rRNA genes (V4 and V5 regions), PCR amplification	Within the PD group, a relative decrease in bacterial taxa associated with health-promoting, anti-inflammatory, neuroprotective, or other beneficial effects on the epithelial barrier, such as *Fusicatenibacter*, was observed. The data confirm the previously reported effects of catechol-O-methyltransferase (COMT) inhibitors on the fecal microbiota of PD patients and suggest a possible effect of L-dopa medication on the relative abundance of several bacterial genera.
11	Wasser et al. (2020) [28]	Cohort study	Huntington’s disease	UHDRS, CAP, HD-CAB, Hopkins Verbal Learning Test-Revised, Symbol Digit Modalities Test, Trail Making Test, Paced tapping, Emotion Recognition Task, OTS of Cambridge	Sequencing of the bacterial 16S rRNA genes	Intestinal microbiome measurements revealed significant differences in the microbial communities between the combined Huntington’s disease gene expansion carrier (HDGEC) group and healthy controls. Major shifts in microbial community structure were also detected at the phylum and family levels, and functional pathways and enzymes that were affected in our HDGEC group were identified. Associations between gut bacteria, cognitive performance, and clinical outcomes were also discovered within the HDGEC group.
12	Vascellari et al. (2020) [35]	Cohort study	Parkinson’s disease	GC-MS analysis	Sequencing of the bacterial 16S rRNA genes (V3 and V4 regions)	The most significant changes within the PD group emphasized a reduction in bacterial taxa associated with anti-inflammatory/neuroprotective effects, particularly in the *Lachnospiraceae* family and its key members, such as *Butyrivibrio*, *Pseudobutyrivibrio*, *Coprococcus*, and *Blautia*. A direct evaluation of fecal metabolites revealed changes in several classes of metabolites. Most of the altered metabolites correlate strongly with the abundance of members of the *Lachnospiraceae* family, suggesting that these gut bacteria correlate with altered metabolic rates in PD.
13	Barone et al. (2021) [42]	Cohort study	Multiple sclerosis	MFIS-5, 6MWT, FFQ, paraclinical tests	Sequencing of the bacterial 16S rRNA genes (V3 and V4 regions), PCR amplification	B-HIPE led to a modulation of MS-typical dysbiosis with reduced concentrations of pathobionts and a replenishment of beneficial short-chain fatty acid producers. This partial restoration of a eubiotic profile may help to counteract the inflammatory tone typically seen in MS, which is supported by reduced circulating lipopolysaccharide levels and decreased populations of pro-inflammatory lymphocytes. Improved physical performance and reduced fatigue were also noted.
14	Hertel et al. (2019) [36]	Cohort study	Parkinson’s disease	UPDRS, mass spectrometry	N/A	The longitudinal trajectory of metabolites was associated with the interconversion of methionine and cysteine via cystathionine, which differed between PD patients and controls. Dopaminergic medication showed strong lipidomic signatures. Taurine-conjugated bile acids correlated with the severity of motor symptoms, while low levels of sulfated taurolithocholate were associated with PD incidence in the general population. Computational modeling predicted changes in sulfur metabolism, driven by *A. muciniphila* and *B. wadsworthia*, which is consistent with the changed metabolome.
15	Hegelmaier et al. (2020) [37]	Case–control study	Parkinson’s disease	UPDRS	Sequencing of the bacterial 16S rRNA genes (V1-V3 regions)	UDPRS III improved significantly and the levodopa-equivalent daily dose decreased after a vegetarian diet and fecal enema in a one-year follow-up. In addition, a significant correlation was observed between the diversity of the gut microbiome and the UPDRS III as well as the abundance of *Ruminococcaceae*. The abundance of *Clostridiaceae* also significantly reduced after an enema.
16	Zeng et al. (2020) [26]	Cohort study	Amyotrophic lateral sclerosis	El Escorial Criteria, UPLC	Sequencing of the bacterial 16S rRNA genes (V4 region), PCR amplification	The analysis showed an apparent change in the microbial structure of ALS patients, with *Bacteroidetes* upregulated at the phylum level and several microbes upregulated at the genus level, while *Firmicutes* were downregulated at the phylum level and *Megamonas* at the genus level compared to healthy controls. In addition, decreased gene function associated with metabolic pathways was observed in ALS patients.
17	Brenner et al. (2017) [40]	Cohort study	Amyotrophic lateral sclerosis	El Escorial Criteria	Quantification of 16S rDNA copy numbers by qRT-PCR, amplification of V3-V6 16S rDNA regions, PiCRUSt	Comparing the 2 groups, the diversity and abundance of the bacterial taxa on the different taxonomic levels as well as PiCRUSt-predicted metagenomes were almost indistinguishable. Significant differences between ALS patients and healthy controls were only observed with regard to the overall number of microbial species (operational taxonomic units) and in the abundance of uncultured *Ruminococcaceae*.
18	Nicholson et al. (2020) [27]	Case–control study	Amyotrophic lateral sclerosis	El Escorial Criteria, ALSFRS-R	Sequencing of the bacterial 16S rRNA genes (V4 region), Illumina HiSeq	The relative abundance of the dominant butyrate-producing bacteria, *Eubacterium rectale* and *Roseburia intestinalis*, was significantly lower in ALS patients compared to HC. These findings lend support to the inference that the gut microbiota could be a risk factor for ALS.
19	Zhou et al. (2021) [39]	Cohort study	Alzheimer’s disease	MMSE, MoCA, CDR, NPI	Sequencing of the bacterial 16S rRNA genes (V3 and V4 regions), PCR amplification, PiCRUSt	The fecal microbial composition of AD patients was quite distinct from that of HC. *Bifidobacterium*, *Sphingomonas*, *Lactobacillus*, and *Blautia* were enriched, while *Odoribacter*, *Anaerobacterium*, and *Papillibacter* were reduced. AD patients with NPS showed decreased abundance of *Chitinophagaceae*, *Taibaiella*, and *Anaerobacterium* compared with those without NPS. Functional pathways were different between AD and HC and between AD patients with and without NPS. A correlation analysis showed that *Sphingomonas* correlated negatively with MMSE; *Anaerobacterium* and *Papillibacter* correlated positively with MMSE and negatively with CDR. *Cytophagia*, *Rhodospirillaceae*, and *Cellvibrio* correlated positively with NPS, while *Chitinophagaceae*, *Taibaiella*, and *Anaerobacterium* correlated negatively with NPS.

## Data Availability

The original contributions presented in the study are included in the article/Supplementary Material, further inquiries can be directed to the corresponding author.

## Supplementary Materials

The following supporting information can be downloaded at https://www.mdpi.com/article/10.3390/microorganisms12081735/s1. Figure S1: Fungal and protist microorganisms related to PD. Figure S2: Bacterial microorganisms related to AD. Figure S3: Bacterial microorganisms related to ALS. Figure S4: Bacterial microorganisms related to MSA. Figure S5: Bacterial microorganisms related to CJD. Figure S6: Bacterial microorganisms related to HD. Figure S7: Bacterial microorganisms related to MS. Table S1: Demographic and clinical parameters of the participants in the included studies. Table S2: All Microbes associated with each Neurodegenerative disorder.
