# Supplementary material for: Gut Microbes Associated with Neurodegenerative Disorders: A Comprehensive Review of the Literature
Source: Microorganisms. 2024 Aug 22;12(8):1735. doi: 10.3390/microorganisms12081735 (PMC11357424; doi:10.3390/microorganisms12081735)
Supplement: Supplementary file 1 [file microorganisms-12-01735-s001.zip › Figure S5.Bacterial microorganisms related to CJD.pdf]

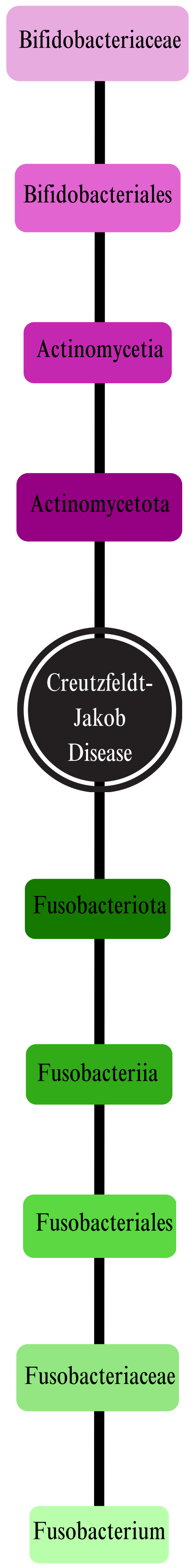
Bifidobacteriaceae
Bifidobacteriales
Actinomycetia
Actinomycetota
Creutzfeldt-Jakob Disease
Fusobacteriota
Fusobacteriia
Fusobacteriales
Fusobacteriaceae
Fusobacterium
