# Supplementary material for: Gut Microbes Associated with Neurodegenerative Disorders: A Comprehensive Review of the Literature
Source: Microorganisms. 2024 Aug 22;12(8):1735. doi: 10.3390/microorganisms12081735 (PMC11357424; doi:10.3390/microorganisms12081735)
Supplement: Supplementary file 1 [file microorganisms-12-01735-s001.zip › Table S1.Demographic and clinical parameters of the participants in the included studies .pdf]

Table S1. Demographic and clinical parameters of the participants in the included studies.

		n	Age (years)	Gender (% m/f)	BMI (kg/m²)	Disease duration (years)	Medication (%)[a]	MMSE scores	Constipation (%)[b]
**Qian et al. (2018)**	PD	45	68.1	48.9%	22.8	5.7	100%	29.0	53.3%
	HC	45	67.9	51.1%	23.4	-	-	-	6.7%
**Li et al. (2022)**	PD	78	65.5	59.0%	23.2	-	-	26.6	-
	HC	75	59.2	36.0%	23.3	-	-	-	-
**Weis et al. (2021)**	PD	18	66.9	66.6%	-	7.4	66.6%	-	27.8%
	HC	18	65.9	44.4%	-	-	-	-	11.1%
**Murros et al. (2021)**	PD	20	70.0	60.0%	25.0	10.0	70.0%	-	70.0%
	HC	20	68.5	40.0%	23.5	-	-	-	0.0%
**Weis et al. (2019)**	PD	34	67.9	67.6%	-	-	70.6%	-	20.6%
	HC	25	63.9	44.0%	-	-	-	-	8.0%
**Vascellari et al. (2020)**	PD	64	71.4	68.8%	26.1	-	100%	-	57.8%
	HC	51	51.7	60.8%	23.7	-	-	-	0.0%
**Hertel et al. (2019)**	PD	30	64.0	50.0%	27.8	1.0	83.3%	-	-
	HC	30	64.4	50.0%	28.0	-	-	-	-
**Hegelmaier et al. (2020)**	PD	54	61.0	50.0%	26.2	9.1	74.1%	-	-
	HC	34	52.8	41.2%	26.1	-	-	-	-
**Liu et al. (2019)**	AD	33	74.9	57.6%	22.0	-	-	17.9	-
	aMCI	32	70.5	43.7%	22.4	-	-	27.3	-
	HC	32	76.9	50.0%	22.2	-	-	28.8	-
**Zhou et al. (2021)**	AD with NPS	30	73.0	36.7%	22.3	-	-	18.5	-
	AD without NPS	30	72.6	43.3%	22.0	-	-	19.4	-
	HC	32	71.1	43.8%	21.7	-	-	28.1	-
**Zeng et al. (2020)**	ALS	20	53.9	60.0%	22.8	-	-	-	-
	HC	20	50.6	60.0%	21.8	-	-	-	-
**Brenner et al. (2017)**	ALS	25	57.6	48.0%	25.6	1.8	-	-	-
	HC	32	56.0	50.0%	25.7	-	-	-	-
**Nicholson et al. (2020)**	ALS	66	57.0	60.6%	-	0.9	62.1%	-	-
	NDGC	12	53.8	41.7%	-	-	8.3%	-	-
	HC	61	54.6	41.0%	-	-	0.0%	-	-
**Wan et al. (2019)**	MSA	15	56.7	53.3%	22.4	2.3	-	26.3	-
	HC	15	53.8	66.7%	22.8	-	-	-	-
**Barone et al. (2021)**	MS	14	49.4	50.0%	-	19.3	-	-	-
**Wasser et al. (2020)**	HD	42	50.2	52.4%	-	5.1	-	-	-
	HC	36	50.6	41.7%	-	-	-	-	-
**Guo et al. (2022)**	CJD	10	57.1	60.0%	23.8	0.8	-	18.7	-
	HC	10	58.2	60.0%	23.4	-	-	-	-
**Raghavan et al. (2023)**	-								

**Zhuang et al. (2020)**	-

Data are shown as mean (SD). Abbreviations: n, number of individuals; m, male; f, female; BMI, body mass index; MMSE, mini-mental state examination; HC, healthy controls; PD, Parkinson's disease; AD, Alzheimer's disease; aMCI, amnestic mild cognitive impairment; NPS, neuropsychiatric symptoms; ALS, amyotrophic lateral sclerosis; NDGC, neurodegenerative controls; MSA, multiple system atrophy; MS, multiple sclerosis; HD, Huntington's disease; CJD, Creutzfeldt-Jacob disease; "-", not available.

[a] The percentage refers to the specific number of patients, receiving a form of medication for their condition, in relation to the total number of patients in each study.
[b] The percentage refers to the specific number of individuals suffering from constipation, in relation to the total number of individuals in each study.
