# Supplementary material for: Gut Microbes Associated with Neurodegenerative Disorders: A Comprehensive Review of the Literature
Source: Microorganisms. 2024 Aug 22;12(8):1735. doi: 10.3390/microorganisms12081735 (PMC11357424; doi:10.3390/microorganisms12081735)
Supplement: Supplementary file 1 [file microorganisms-12-01735-s001.zip › Table S2.All Microbes associated with each Neurodegenerative disorder.pdf]

Table S2. All Microbes associated with each Neurodegenerative disorder

Parkinson's Disease

Phylum	Class	Order	Family	Genus	Species	
**Bacteria**						
Actinomycetota	Actinomycetia	Bifidobacteriales	Bifidobacteriaceae	Bifidobacterium		
Bacteroidetes	Bacteroidia	Bacteroidales	Odoribacteraceae	Odoribacter		
Bacteroidetes	Bacteroidia	Bacteroidales	Prevotellaceae	Prevotella		
Bacteroidetes	Bacteroidia	Bacteroidales	Rikenellaceae	Alistipes		
Cyanobacteria	Cyanophyceae	Nostocales	Aphanizomenonaceae	Dolichospermum		
Firmicutes	Clostridia	Eubacteriales	Lachnospiraceae	Blautia		
Firmicutes	Clostridia	Eubacteriales	Lachnospiraceae	Butyrivibrio		
Firmicutes	Clostridia	Eubacteriales	Lachnospiraceae	Clostridium IV		
Firmicutes	Clostridia	Eubacteriales	Lachnospiraceae	Clostridium XI		
Firmicutes	Clostridia	Eubacteriales	Lachnospiraceae	Clostridium XVIII		
Firmicutes	Clostridia	Eubacteriales	Lachnospiraceae	Coprococcus		
Firmicutes	Clostridia	Eubacteriales	Lachnospiraceae	Dorea		
Firmicutes	Clostridia	Eubacteriales	Lachnospiraceae	Fusicatenibacter		
Firmicutes	Clostridia	Eubacteriales	Lachnospiraceae	Roseburia		
Firmicutes	Clostridia	Eubacteriales	Ruminococcaceae	Anaerotruncus		
Firmicutes	Clostridia	Eubacteriales	Ruminococcaceae	Butyricicoccus		
Firmicutes	Clostridia	Eubacteriales	Ruminococcaceae	Faecalibacterium		
Firmicutes	Erysipelotrichia	Erysipelotrichales	Erysipelotrichaceae	Holdemania		
Firmicutes	Negativicutes	Acidaminococcales	Acidaminococcaceae	Phascolarctobacterium		
Firmicutes	Tissierellia	Tissierellales	Peptoniphilaceae	Finegoldia		
Firmicutes	Tissierellia	Tissierellales	Peptoniphilaceae	Peptoniphilus		
Pseudomonadota	Alphaproteobacteria	Sphingomonadales	Sphingomonadaceae	Sphingomonas		
Pseudomonadota	Betaproteobacteria	Burkholderiales	Comamonadaceae	Aquabacterium		
Thermodesulfobacteriota	Desulfovibrionia	Desulfovibrionales	Desulfovibrionaceae	Bilophila	Bilophila wadsworthia	
Thermodesulfobacteriota	Desulfovibrionia	Desulfovibrionales	Desulfovibrionaceae	Desulfovibrio		
Verrucomicrobiota	Verrucomicrobiae	Verrucomicrobiales	Akkermansiaceae	Akkermansia	Akkermansia muciniphila	

Fungi and Protists						
Gyrista	Synurophyceae	Ochromonadales	Ochromonadaceae	Poterioochromonas		prostist
Ascomycota	Saccharomycetes	Saccharomycetales	Dipodascaceae	Geotrichum		fungus
Cercozoa	Sarcomonadea	Cercomonadida	Heteromitidae	Heteromita		prostist
Cercozoa	Sarcomonadea	Cercomonadida	Cercomonadidae	Cercomonas		prostist
Zooflaellates						prostist

Alzheimer's Disease

Phylum	Class	Order	Family	Genus	Species	
Bacteroidetes	Bacteroidia	Bacteroidales	Odoribacteraceae	Odoribacter		
Firmicutes	Clostridia	Eubacteriales	Clostridiaceae			
Firmicutes	Clostridia	Eubacteriales	Eubacteriaceae	Eubacterium		
Firmicutes	Clostridia	Eubacteriales	Lachnospiraceae	Blautia		
Firmicutes	Clostridia	Eubacteriales	Ruminococcaceae	Anaerobacterium		
Firmicutes	Clostridia	Eubacteriales	Ruminococcaceae	Papillibacter		
Firmicutes	Clostridia	Eubacteriales	Ruminococcaceae	Ruminococcus		
Pseudomonadota	Gammaproteobacteria	Enterobacteriales	Enterobacteriaceae	Escherichia	Escherichia coli	

Amyotrophic Lateral Sclerosis

Phylum	Class	Order	Family	Genus	Species	
Bacteroidetes						
Firmicutes	Clostridia	Eubacteriales	Eubacteriaceae	Eubacterium	Eubacterium rectale	
Firmicutes	Clostridia	Eubacteriales	Lachnospiraceae	Roseburia	Roseburia intestinalis	
Firmicutes	Clostridia	Eubacteriales	Ruminococcaceae			
Firmicutes	Negativicutes	Selenomonadales	Selenomonadaceae	Megamonas		

Multiple System Atrophy

Phylum	Class	Order	Family	Genus	Species	
Actinomycetota	Actinomycetia	Bifidobacteriales	Bifidobacteriaceae	Bifidobacterium		
Bacteroidetes	Bacteroidia	Bacteroidales	Bacteroidaceae	Phocaeicola	Phocaeicola coprocola	
Bacteroidetes	Bacteroidia	Bacteroidales	Bacteroidaceae	Phocaeicola	Phocaeicola plebeius	
Firmicutes	Bacilli	Bacillales	Staphylococcaceae	Staphylococcus	Staphylococcus xylosus	
Firmicutes	Clostridia	Eubacteriales	Lachnospiraceae	Roseburia	Roseburia hominis	
Firmicutes	Clostridia	Eubacteriales	Lachnospiraceae	Blautia		
Firmicutes	Clostridia	Eubacteriales	Lachnospiraceae	Tyzzerella	Clostridium nexile	
Firmicutes	Negativicutes	Selenomonadales	Selenomonadaceae	Megamonas		
Firmicutes	Negativicutes	Selenomonadales	Selenomonadaceae	Megamonas	Megamonas funiformis	
Pseudomonadota	Gammaproteobacteria	Pasteurellales	Pasteurellaceae	Aggregatibacter		
Verrucomicrobiota	Verrucomicrobiae	Verrucomicrobiales	Akkermansiaceae	Akkermansia	Akkermansia muciniphila	

Creutzfeldt Jacob Disease

Phylum	Class	Order	Family	Genus	Species	
Actinomycetota	Actinomycetia	Bifidobacteriales	Bifidobacteriaceae			
Fusobacteriota	Fusobacteriia	Fusobacteriales	Fusobacteriaceae	Fusobacterium		

Huntington’s Disease

Phylum	Class	Order	Family	Genus	Species	
Bacteroidetes						
Firmicutes	Clostridia	Eubacteriales	Lachnospiraceae			
Verrucomicrobiota	Verrucomicrobiae	Verrucomicrobiales	Akkermansiaceae			

Multiple Sclerosis

Phylum	Class	Order	Family	Genus	Species	
Actinomycetota	Coriobacteriia	Coriobacteriales	Coriobacteriaceae	Collinsella		
Actinomycetota	Coriobacteriia	Eggerthellales	Eggerthellaceae	Eggerthella		
Bacteroidetes	Bacteroidia	Bacteroidales	Prevotellaceae	Prevotella		
