# Supplementary figures and images for: Gut Microbes Associated with Neurodegenerative Disorders: A Comprehensive Review of the Literature
Source: Microorganisms. 2024 Aug 22;12(8):1735. doi: 10.3390/microorganisms12081735 (PMC11357424; doi:10.3390/microorganisms12081735)

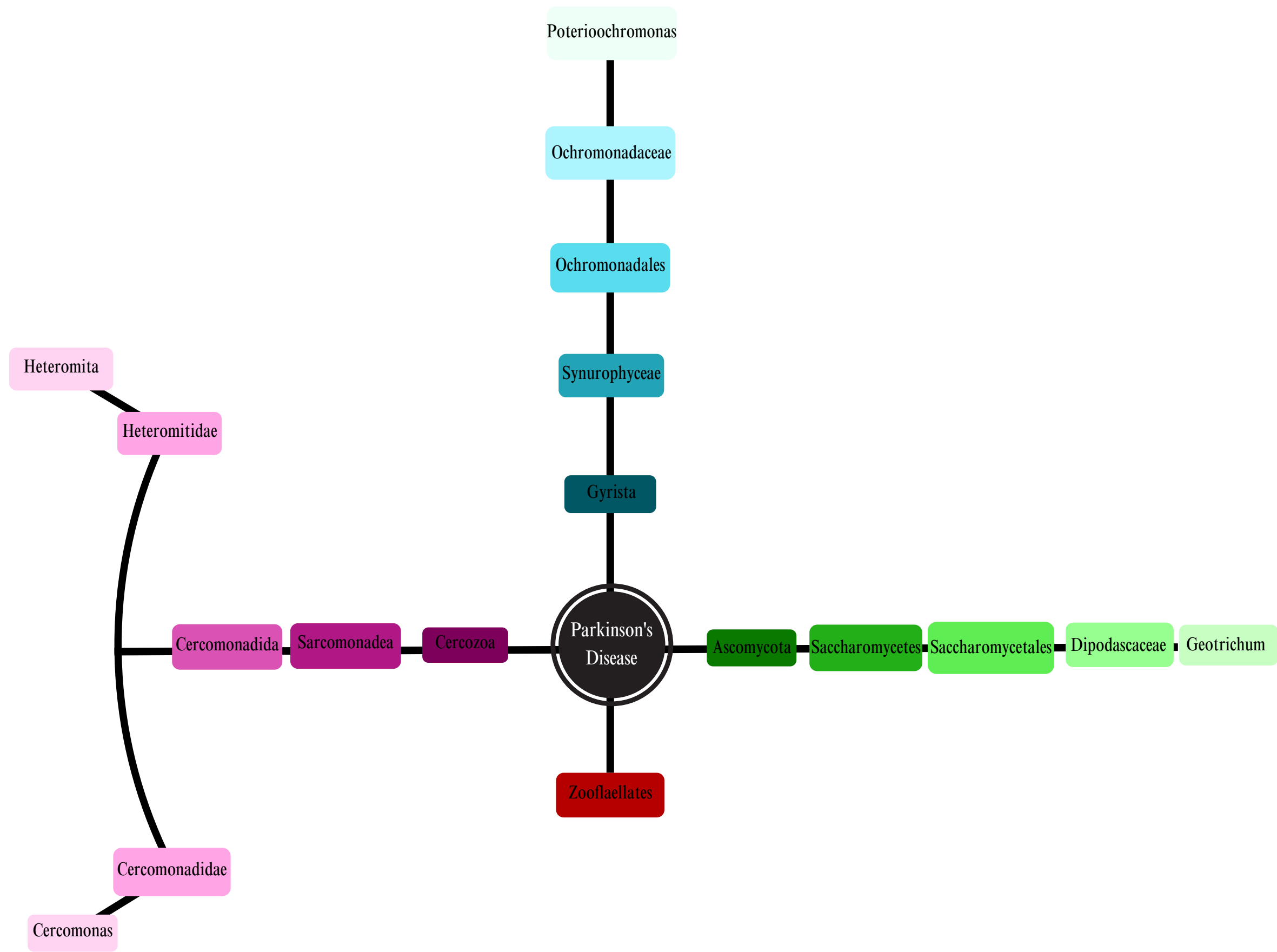

Supplement: Supplementary file 1 [file microorganisms-12-01735-s001.zip › Figure S1.Fungal and protist microorganisms related to PD.pdf]

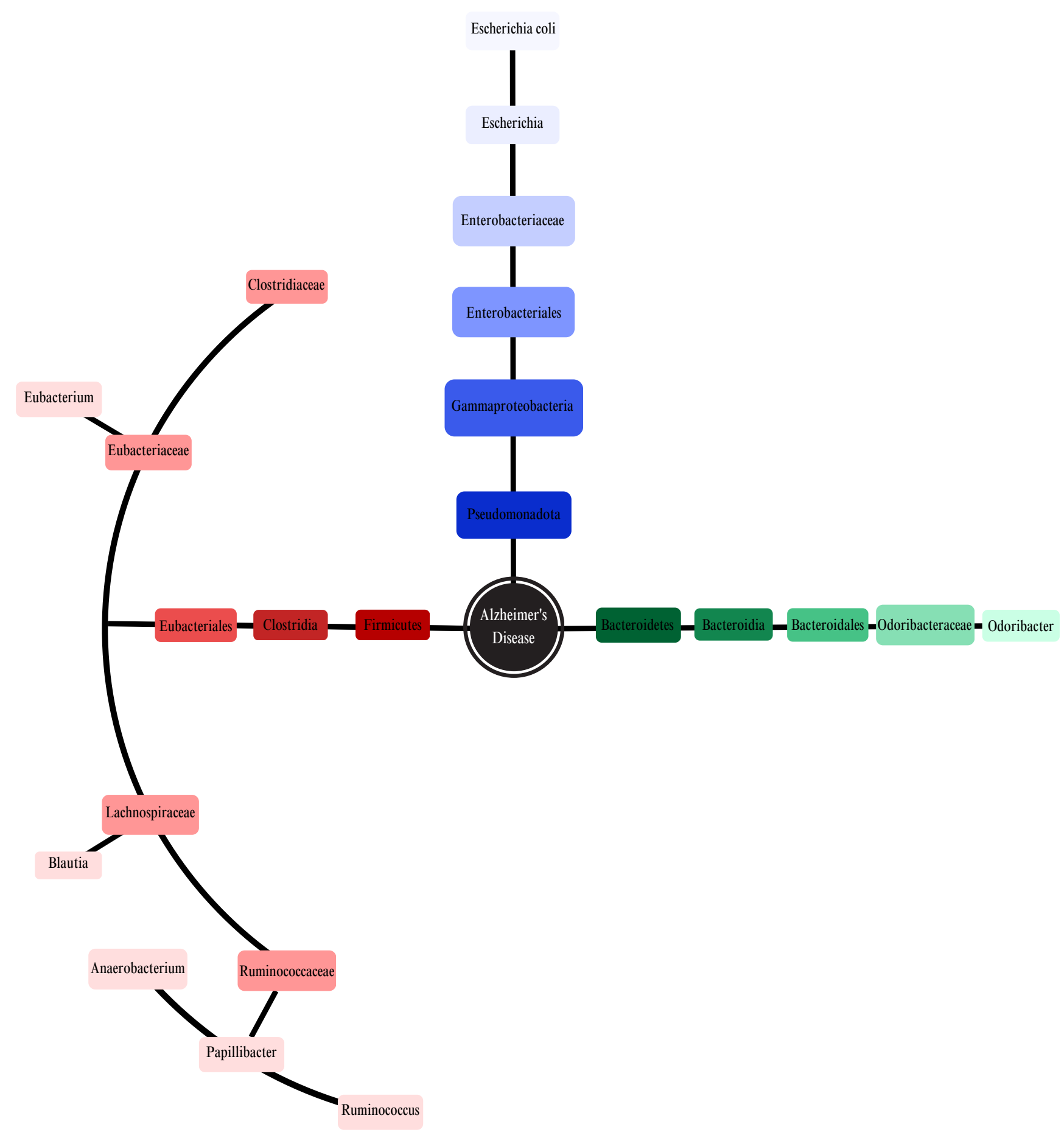

Supplement: Supplementary file 1 [file microorganisms-12-01735-s001.zip › Figure S2.Bacterial microorganisms related to AD.pdf]

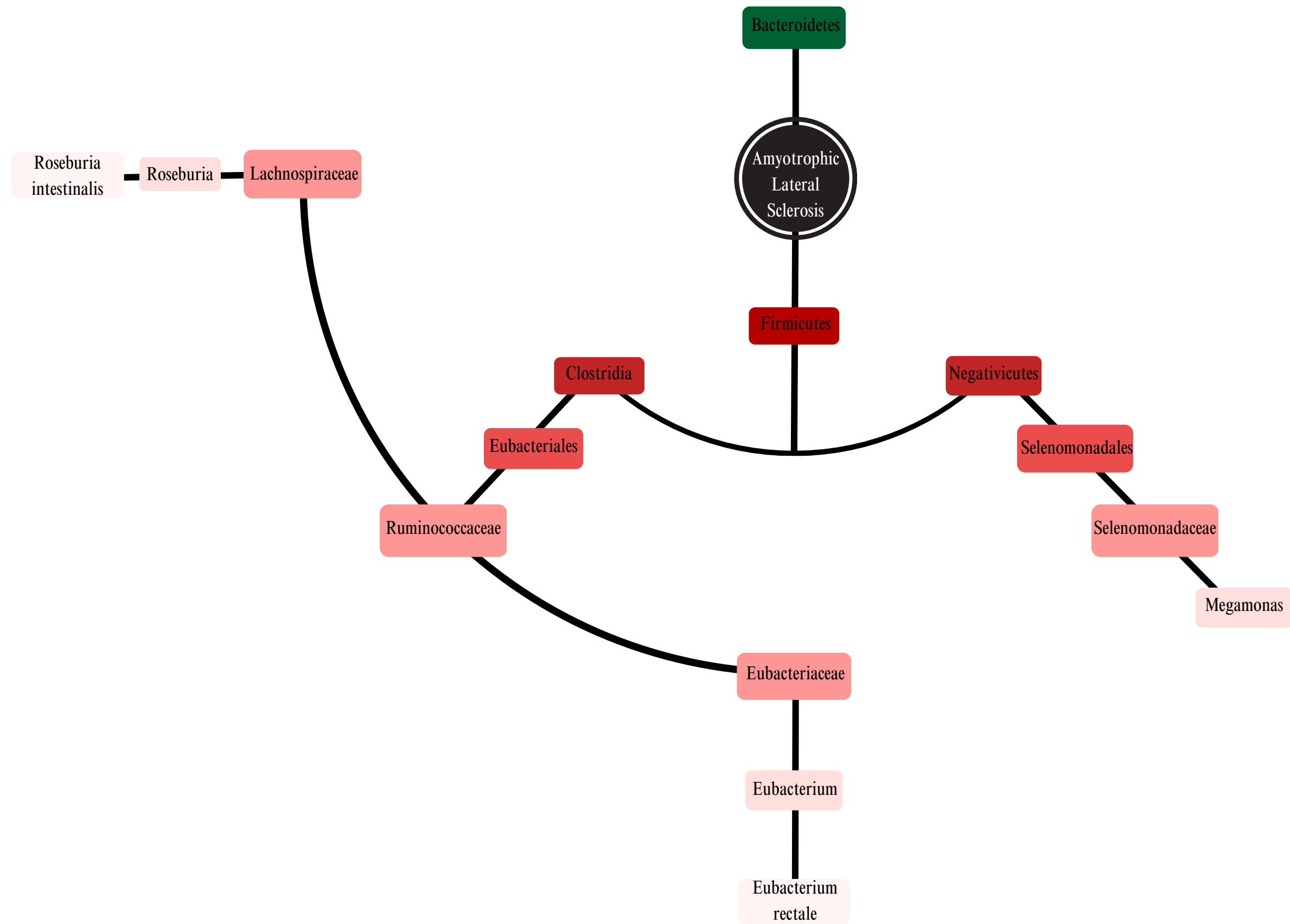

Supplement: Supplementary file 1 [file microorganisms-12-01735-s001.zip › Figure S3.Bacterial microorganisms related to ALS. pdf.pdf]

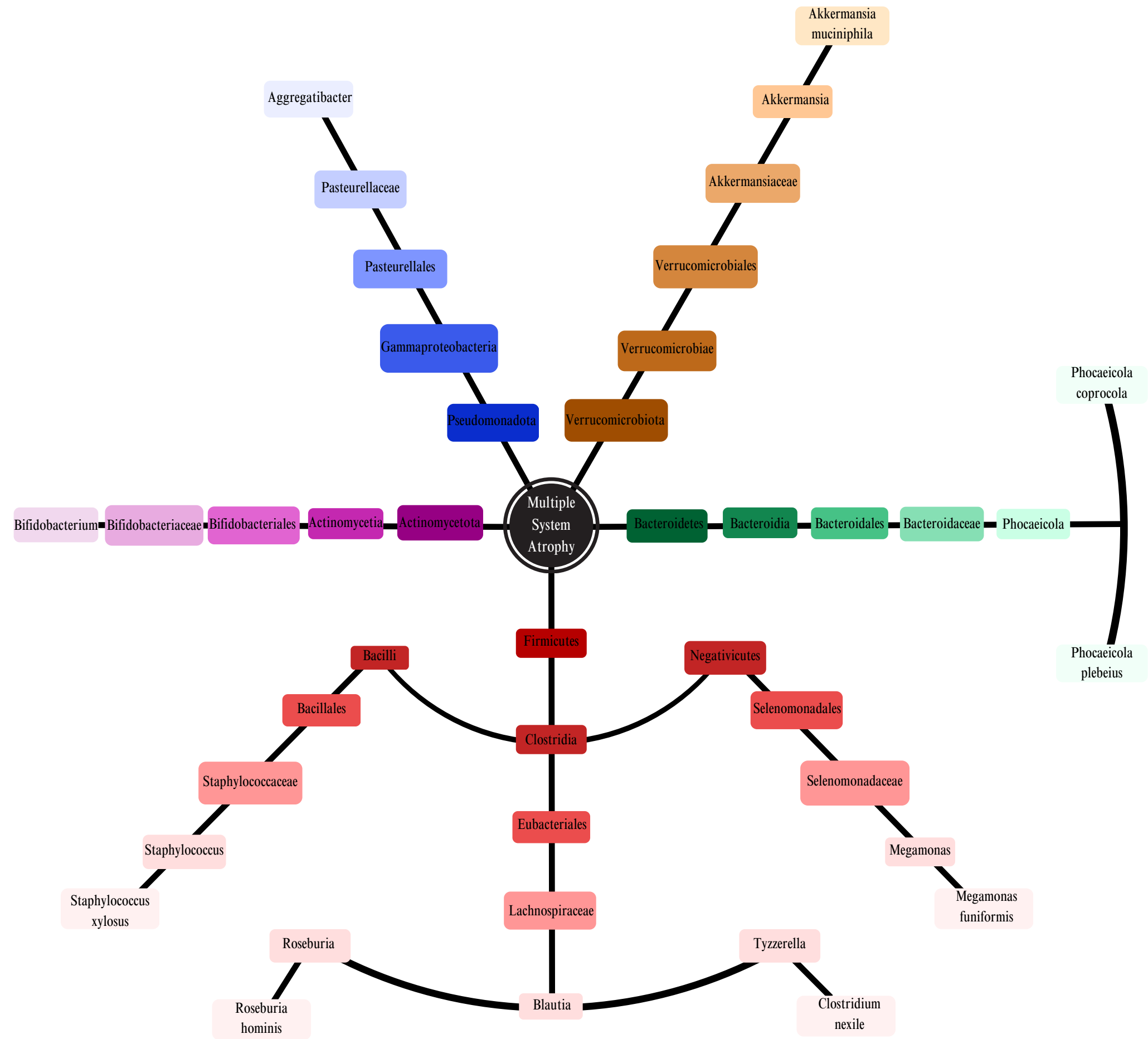

Supplement: Supplementary file 1 [file microorganisms-12-01735-s001.zip › Figure S4.Bacterial microorganisms related to MSA. pdf.pdf]

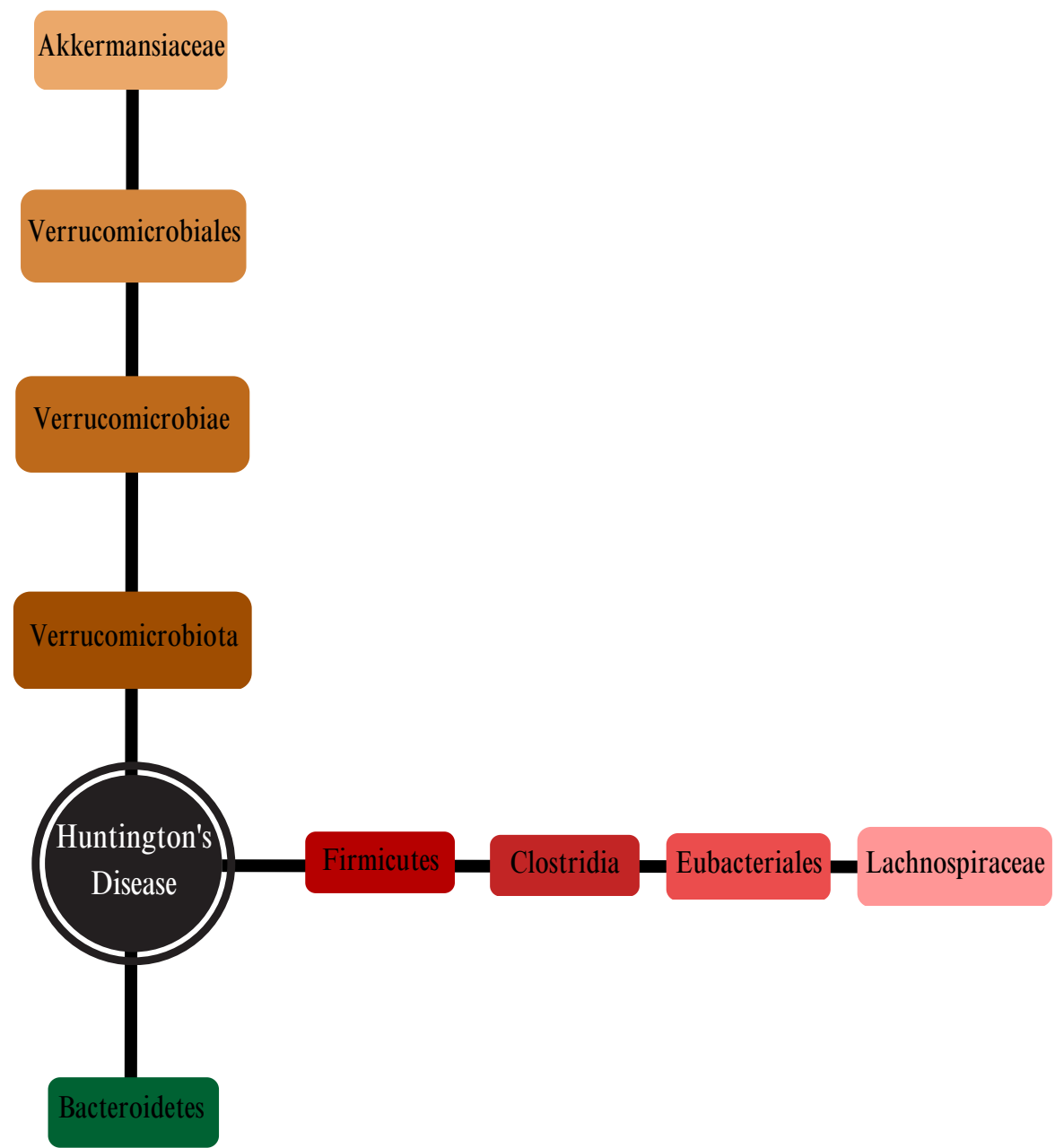

Supplement: Supplementary file 1 [file microorganisms-12-01735-s001.zip › Figure S6.Bacterial microorganisms related to HD. pdf.pdf]

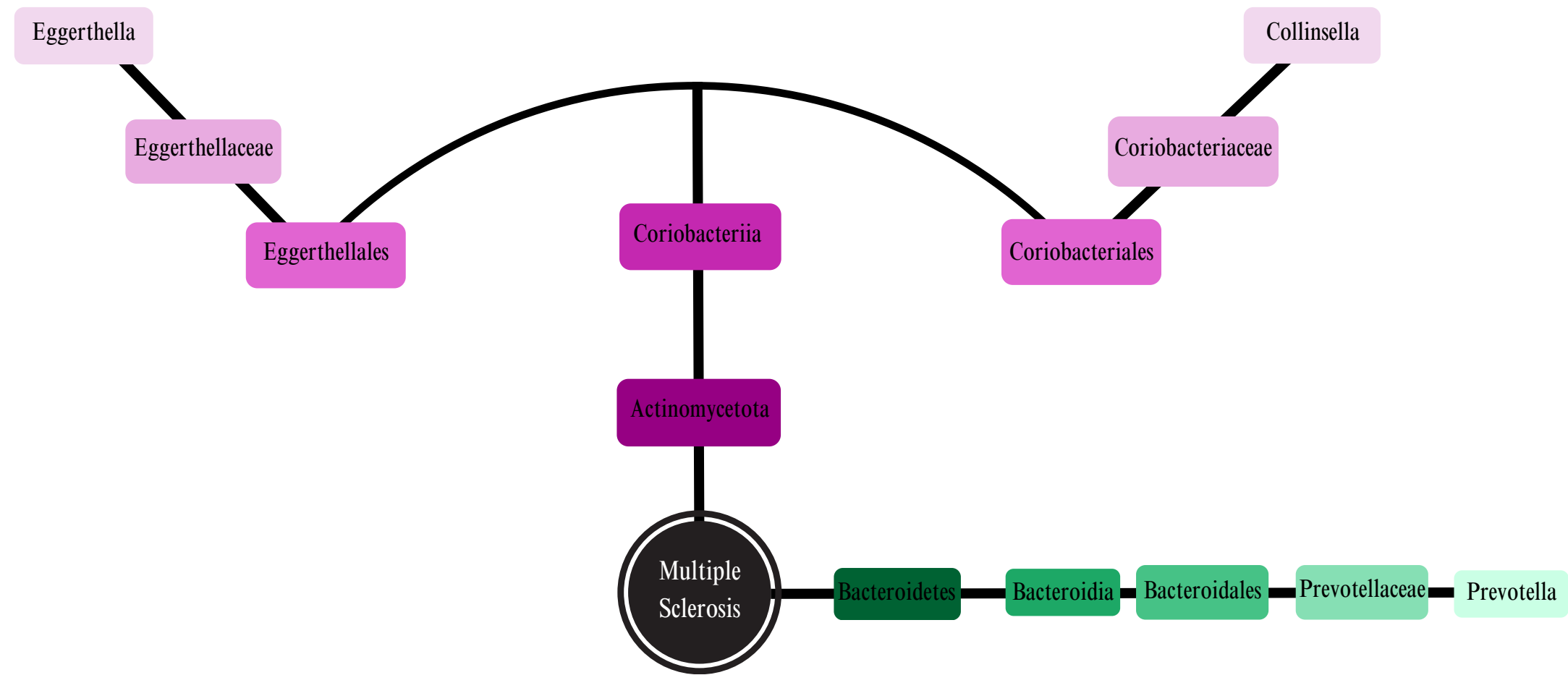

Supplement: Supplementary file 1 [file microorganisms-12-01735-s001.zip › Figure S7.Bacterial microorganisms related to MS.pdf]
